# Bilateral Modified Thoracoabdominal Nerve Block Through a Perichondrial Approach in Patients Undergoing Major Abdominal Surgery: A Randomized Single-Blind Controlled Trial

**DOI:** 10.4274/TJAR.2025.241752

**Published:** 2025-02-11

**Authors:** Beliz Bilgili, Ecem Güçlü Öztürk, Gamze Tanırgan Çabaklı, Gülşen Cebecik Teomete, Merve Ergenç

**Affiliations:** 1 Marmara University Faculty of Medicine, Department of Anaesthesiology and Reanimation, İstanbul, Türkiye; 2 Marmara University Pendik Training and Research Hospital, Clinic of Anaesthesiology and Reanimation, İstanbul, Türkiye

**Keywords:** Analgesia, major abdominal surgery, plane block, pain management, postoperative pain, ultrasonography

## Abstract

**Objective:**

Modified thoracoabdominal nerve block with a perichondrial approach (M-TAPA) provides effective analgesia in the anterior and lateral thoracoabdominal regions. Previous studies have shown the efficacy of M-TAPA in laparoscopic surgery. The primary aim of this study was to investigate the efficacy of M-TAPA block in patients undergoing open major abdominal surgery.

**Methods:**

This study was designed as a prospective, randomized, single-blind, controlled study. A total of 43 patients were included in the study. In group M-TAPA, the block was performed bilaterally at the end of the surgery. Local wound infiltration was performed on the control group. Postoperative analgesia was provided with patient-controlled intravenous morphine. When numeric rating scale (NRS) pain scores exceeded 4, rescue analgesia with tramadol was administered. The primary outcome of this study was to compare the 24-hour total morphine consumption. The secondary outcomes included comparing pain scores, rescue analgesia requirements, and patient satisfaction.

**Results:**

Regarding our primary outcome, median morphine consumption during the first 24 hours was lower in the M-TAPA group [16 (14-18)] than in the control group [24.5 (19.5-27)] (*P* < 0.01, 95% confidence interval: -9, 42 and -3.01). Additionally, NRS scores were significantly lower and patient satisfaction was significantly higher in the M-TAPA group. The need for rescue analgesics in the first 24 hours was comparable among the study groups.

**Conclusion:**

The M-TAPA block is an effective abdominal wall block that can be considered part of multimodal analgesia in open major abdominal surgery.

**Main Points**

• Major abdominal surgery is associated with severe postoperative pain.

• The use of multimodal analgesic therapy is essential for postoperative pain management in major abdominal surgery.

• The Modified thoracoabdominal nerve block with a perichondrial approach (M-TAPA) block has the potential to offer extensive analgesia across a wide area of the anterior abdomen with a single puncture per side, indicating promise for effective pain relief.

• Bilateral M-TAPA block can be used as part of multimodal analgesia in open major abdominal surgery with a midline incision.

## Introduction

Intense postoperative pain and distress are linked to increased morbidity, prolonged recovery time, and decreased patient satisfaction. Inadequate pain management is a risk factor for ongoing opioid use and persistent postoperative pain.^1^ While traditional opioid-based regimens are effective, they have adverse effects that can hinder recovery and prolong hospital stays. In open abdominal surgeries, enhanced recovery protocols emphasize limiting opioid use through multimodal analgesia, including central and peripheral nerve blocks.^2, 3^ Despite being effective, central blocks are less frequently used due to technical challenges and systemic side effects. Peripheral blocks, which are easier to administer, offer targeted pain relief with fewer systemic effects, thereby reducing opioid consumption and related side effects.^4, 5^

Major abdominal surgery encompasses a wide range of procedures across diverse patient groups, resulting in varying levels of pain and analgesia needs. Fascial plane blocks offer effective analgesia for abdominal surgeries, thereby avoiding the sympathetic block and hypotension associated with central blocks, as well as the risk of epidural hematoma in patients with coagulopathy. Recent studies have demonstrated the efficacy of quadratus lumborum blocks, erector spinae plane blocks, transversus abdominis plane, and rectus sheath blocks in providing postoperative analgesia and assisting in functional recovery in major abdominal surgery.^6, 7, 8, 9, 10^ Consequently, interest in local anaesthetic abdominal wall blocks as a component of multimodal analgesia has increased.

Modified thoracoabdominal nerve block through the pericondrial approach (M-TAPA), is a novel plane block technique that provides analgesic effects through the injection of local anaesthetic at the lower aspect of the chondrium, at the costochondral corner.^11^ Studies have demonstrated the analgesic effects of the M-TAPA block in the T7-11, T5-10, and T3-12 dermatomes.^11, 12, 13^ Cadaveric evaluations have shown that dye spreads in T 8-11.^14^ The M-TAPA block has the potential to offer extensive analgesia across a wide area of the anterior abdomen with a single puncture per side, indicating promise for effective pain relief. However, further investigation is needed, particularly in laparotomies.

Therefore, this prospective randomized study aimed to evaluate the efficacy of ultrasound-guided bilateral M-TAPA block for pain management compared with surgical site infiltration in patients who underwent open major abdominal surgery. We hypothesized that bilateral M-TAPA would provide superior analgesia compared with surgical site infiltration. The primary outcome of this study was the comparison of 24-hour total morphine consumption. The secondary outcomes included postoperative pain scores, the need for rescue analgesia, and patient satisfaction.

## Methods

### Study Design

This single-center, prospective, and randomized study was reported in accordance with the Consolidated Standards of Reporting Trials (CONSORT) guidelines and was approved by the Marmara University Faculty of Medicine Clinical Research Ethics Committee (protocol code: 03.3.2023/371, date: 03.03.2023). It was conducted in compliance with the Ethical Principles for Medical Research Involving Human Subjects, as outlined in the Helsinki Declaration, and was registered prior to patient enrollment at ClinicalTrials.gov (NCT06384677).

### Eligibility Criteria

Between April 2023 and February 2024, patients older than 18 years who were scheduled for elective primary open abdominal surgery under general anaesthesia (GA) and who provided informed consent were included in the study. Exclusion criteria were allergy to local anaesthetics, infection at the block site, refusal of the procedure, prior gabapentin use, revision or emergency surgery, planned or unexpected postoperative admission to the intensive care unit (ICU), and inability to use the patient-controlled analgesia (PCA) device due to cognitive impairment like postoperative delirium.

### Randomization

**Figure 1:**
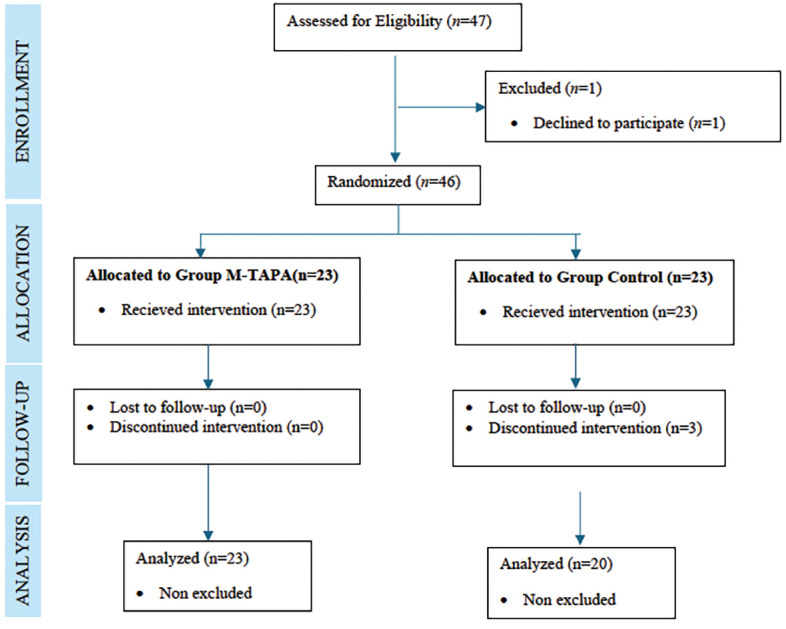
CONSORT flowchart

A simple randomization method was utilized, with allocation concealment achieved by randomly drawing from sealed opaque envelopes containing assignments the day before surgery. These envelopes were attached to the preanaesthetic assessment form by the investigator. The attending anaesthetist opened the envelope after the patient was under GA, ensuring patient blinding. If surgery was cancelled, or the patient could not use PCA postoperatively, the envelope was returned to the pool. A CONSORT diagram illustrates the participant flow (Figure 1).

### Intervention

All surgeries were performed by the same surgical team using a midline incision above and below the umbilicus. Standard monitoring, including electrocardiography, invasive blood pressure measurements, and peripheral oxygen saturation, was initiated. After the patients were placed under GA and maintained with intravenous remifentanil infusion and sevoflurane inhalation, the envelopes were opened. At the end of the surgery, the treatment group received a block performed by designated anaesthetists, who had extensive experience, using a linear (12-4 MHz) ultrasound probe (Sparq Ultrasound; Philips, USA) with the patients in the supine position. In the control group, the surgeons administered local infiltration to the surgical site.

For the M-TAPA block, the 10^th^ costal cartilage was identified by locating the notch between the 9^th^ and 10^th^ costal cartilages. The transducer was then placed on the chondrium in the sagittal plane at the level of the 9^th^-10^th^ ribs. The probe was angled deeply to visualize the lower aspect of the costochondrium. A 100 mm, 21 G needle (Sonoplex^®^ Pajunk Medizintechnologie, Germany) was inserted. After confirming negative aspiration, 20 mL of 0.25% bupivacaine was injected per side between the transversus abdominis muscle and the lower aspect of the costal cartilage. In the control group, the surgeon performed local infiltration with 40 mL of 0.25% bupivacaine around the incisions.

At the end of surgery, the routine analgesia protocol in both groups included intravenous morphine (0.1 mg kg^-1^) and intravenous paracetamol (1 g). Postoperative analgesia was managed via PCA with intravenous morphine administered at no basal rate, with a 1 mg bolus and a 10-minute lockout period, hourly limit were set at 6 mg h^-1^, which continued for 48 hours after surgery. Additionally, all patients received 1000 mg of intravenous paracetamol every six hours. Demographic and baseline characteristics, as well as surgical details, were documented. Postoperative pain was assessed using the numerical rating scale (NRS)-from 0 (no pain) to 10 (worst pain imaginable)-at the 0, 2^nd^, 6^th^, 12^th^, 24^th^, 36^th^, and 48^th^ hours. If the NRS score was ≥4, intravenous tramadol (1 mg kg^-1^) was administered as rescue analgesia. An anaesthesiologist conducted pinprick tests to assess the sensory block in the M-TAPA group after the first hour of surgery, to ensure block success, without the involvement of the anaesthesiologist who performed the M-TAPA block. A postoperative pain team, blinded to the study conditions, recorded all relevant data, including total morphine consumption, pain scores, rescue analgesic requests, and adverse effects such as nausea, vomiting, and itching. Hemodynamic data were also collected. Patient satisfaction status was classified as complaining, dissatisfied, satisfied, very satisfied on a scale from 1 to 4.

### Statistical Analysis

The data were collected by the investigator and analyzed with Number Cruncher Statistical System (NCSS) 2020 statistical software (NCSS LLC, Kaysville, Utah, USA). For the evaluation of the study data, quantitative variables, including the mean, standard deviation, median, minimum, and maximum values, were presented via descriptive statistical methods. Qualitative variables are displayed as frequencies and percentages. The Shapiro-Wilks test and box plot graphics were employed to assess the normality of the data distribution. Student’s t-test was used for normally distributed quantitative comparisons between two groups, whereas the Mann-Whitney U test was applied for non-normally distributed variables. For non-normally distributed group comparisons, the Friedman test and Wilcoxon-corrected pairwise comparisons were used. The chi-square test and Fisher’s exact test were used for qualitative data comparisons. The results were considered significant at a 95% confidence interval, with *P *< 0.05.

### Sample Size Estimation

The sample size was estimated based on a preliminary study from our center with ten patients (unpublished). In this initial study, the mean morphine consumption in the first 24 hours after surgery was 17.4 mg (±8.5) for the M-TAPA group and 28.3 mg (±16.9) for the surgical site infiltration group. The effect size was calculated as 0.814. Using G^*^Power 3.1, we determined that at least 20 patients per group were needed to achieve a power of 0.80, an alpha level of 0.05, and an effect size of 0.85. To account for a 15% drop-out rate, the study planned to include 23 patients in each group.

## Results

A total of 47 patients were initially assessed for eligibility. One patient refused to participate, leaving 46 patients who were included in the randomization. M-TAPA was successfully performed on 23 patients in the M-TAPA group without any complications. Three patients’ follow-ups were discontinued due to ICU admission for two patients and the development of postoperative delirium for one patient. The CONSORT flow chart of the study is provided in Figure 1.^15^

The demographic characteristics of the patients, including sex, body mass index, American Society of Anesthesiology status, comorbidities, and type of surgery, were comparable between the two groups. The mean age of the patients was 59.23±11.92 years. The most common surgical operation was the Whipple procedure (53.5%). The study groups were comparable in terms of intravenous fluids and blood products, as well as the durations of surgery and anaesthesia (Table 1). In the M-TAPA group, all patients maintained bilateral sensory block in the T8-11 dermatomal distribution.

In terms of our primary outcome, median morphine consumption during the first 24 hours was lower in the M-TAPA group [16 (14-18)] than in the control group [24.5 (19.5-27)] [*P *< 0.01, 95% confidence interval (CI): -9.42 and -3.01]. The M-TAPA group consumed significantly less morphine at all time intervals except for the 2^nd^-6^th^ second to sixth hour (Table 2).

The median NRS score was 4 in the control group at the 6^th^ and 12^th^ hours, which was significantly higher than that in the M-TAPA group (*P*=0.002, 95% CI: -1.54 -0.48; *P* < 0.001, 95% CI: -2.12-0.93, respectively). The median NRS score remained below 4 for both study groups at all time intervals, except the 6^th^ and 12^th^ hours. NRS scores were significantly lower in the M-TAPA group than in the control group at all time intervals except at the 0^th^ and 48^th^ hour (Table 3).

In the first 2 hours after surgery, 8% of the patients in the M-TAPA group and 30% of those in the control group required rescue analgesia. This ratio was 17% versus 45% between the 2^nd^ and 6^th^ hours, and 4% versus 20% between the 6^th^ and 12^th^ hours. No patient in the M-TAPA group required rescue analgesia between the 12^th^ and 48^th^ hours. The need for rescue analgesics in the first 24 hours was proportionally lower in the M-TAPA group. However, these differences were comparable between the groups over the entire period (Table 4). Patient satisfaction data for the groups are presented in Table 5. Patient satisfaction was significantly greater in the M-TAPA group than in the other groups at all time points.

## Discussion

This prospective, randomized controlled study evaluated the analgesic efficacy of the M-TAPA block in major abdominal surgery. We demonstrated that the bilateral M-TAPA block, performed under ultrasound guidance, significantly reduced morphine consumption and resulted in lower NRS scores at all time points than did the control group. Additionally, the M-TAPA block positively impacted patient satisfaction.

Since the block was first described in 2019, several studies have demonstrated the analgesic effect of M-TAPA and compared it with different plane blocks, mostly in laparoscopic surgeries such as cholecystectomy or hernia repair.^16, 18^ This is the first randomized controlled study on the analgesic efficacy of the M-TAPA block in open major abdominal surgery involving a midline incision above and below the umbilicus.

In this study, M-TAPA block significantly reduced postoperative morphine consumption after surgery. In a study demonstrating the analgesic efficacy of the M-TAPA block, in laparoscopic cholecystectomy, postoperative total tramadol consumption was recorded. This randomized controlled study found that a bilateral M-TAPA block reduced total opioid consumption for up to 24 hours 18. Another study comparing oblique subcostal TAP block with M-TAPA block for postoperative analgesia in patients undergoing laparoscopic cholecystectomy reported that opioid consumption in the first 24 hours was lower in the M-TAPA group.^19^ Several other studies, including patients who had undergone laparoscopic surgeries, reported effective postoperative pain control with M-TAPA block.^12, 13, 16, 20^

While M-TAPA is known to provide effective pain relief in laparoscopic surgery, there is limited evidence regarding its effectiveness in relieving pain during open abdominal surgery. At the time of this trial, only one study in the literature reported the application of the M-TAPA block in open abdominal surgery. In that prospective, observational pilot study, 10 patients who underwent open radical hysterectomy via a vertical incision or laparotomy using a midline incision from under the xiphoid process to the symphysis pubis were included. This case series showed that the analgesic effect lasted up to 24 hours; however, the incisions in the surgeries within the case series were not the same.^14^ In our study, all patients underwent a midline surgical incision above and below the umbilicus.

Although 24-hour total morphine consumption was lower in the M-TAPA group, this difference was not observed between 2^nd^-6^th^ hours. We believe that the absence of variation in morphine consumption between the 2^nd^ and 6^th^ hours may be related to the consumption of rescue analgesics. Although the difference in rescue analgesic requirement is not statistically significant ,the control group has a higher percentage of rescue analgesic requirements compared to the M-TAPA group.

Ohgoshi et al.^21^ investigated the efficacy of continuous M-TAPA block in major abdominal surgery in a case series of two patients. Both patients underwent emergency surgery to remove the adhesion. After induction of GA, M-TAPA block, and catheterization were performed. Neither of the patients required additional analgesics other than PCA in the postoperative period.

In this study, we evaluated NRS scores and patient satisfaction to assess the effectiveness of pain control. The median NRS score was always less than 4 in the M-TAPA group. In the control group, the median NRS scores at the 6^th^ and 12^th^ hours were 4 and 4, respectively. Patients who received the M-TAPA block were more satisfied than patients who did not receive the block. In a randomized controlled study by Bilge et al.^17^ on laparoscopic cholecystectomy, the M-TAPA group had significantly lower NRS scores than did the control group. Alver et al.^16^ reported similar results in patients who underwent laparoscopic hernia repair. A study comparing M-TAPA block with local wound infiltration for postoperative analgesia in patients undergoing laparoscopic cholecystectomy showed that NRS scores were significantly lower in the M-TAPA group during the postoperative hours 1-4 and 5-16, first 4, 20, 16 postoperative hours 20. In a case series of M-TAPA block in patients undergoing open gynecologic surgery, 80% of patients did not require additional analgesics, and patient satisfaction was rated as 8.5 out of 10 (range 8-10)^14^. In a randomized controlled study demonstrating the efficacy of the M-TAPA block during laparoscopic cholecystectomy, patient satisfaction was evaluated with a Likert scale. The Likert score was significantly higher in the M-TAPA group than in the control group.

The need for rescue analgesia is comparable between groups over the entire period. These findings may be due to the effect of multimodal analgesia and the relatively small sample size.

The strengths of our study are as follows. In our institution, the regional anaesthesia team and the pain team are made up of different doctors; as a result, pain intensity and patient satisfaction data are free of bias.

### Study Limitations

This study has several limitations. First, the single-center design may affect the generalizability of the findings. Additionally, while the pinprick test was essential for evaluating the block’s efficacy, it may have compromised patient blinding. We collected NRS scores only when the patient was at rest, not during movement or coughing. Movement-based assessments were excluded from data collection because of their variation based on the surgical differences among patients. Instead of using a validated scoring system for patient satisfaction, we chose to use a simple satisfaction scale based on clinical experience, which was more subjective. Also, the small sample size limits the generalizability of our findings.

Our patient groups that underwent abdominal surgery were highly diverse. More specific patient populations could be used in future studies. In addition, prolonging the duration of the block by placing a catheter may be considered. From a future perspective, multicenter, double-blind, randomized studies comparing M-TAPA with sham blocks could be planned.

## Conclusion

This study demonstrated that M-TAPA block reduces postoperative morphine consumption and NRS scores in patients undergoing open abdominal surgery, with a midline incision above and below the umbilicus. M-TAPA block is an effective abdominal wall block and can be considered part of multimodal analgesia in open major abdominal surgery.

## Ethics

**Ethics Committee Approval:** This study approved by the Marmara University Faculty of Medicine Clinical Research Ethics Committee (protocol code: 03.3.2023/371, date: 03.03.2023).

**Informed Consent:** Written informed consent was obtained from each patient.

## Figures and Tables

**Table 1. Demographic and Peroperative Informations of the Patients:** 

-		**Analgesia group**		***P***
		**M-TAPA (n=23)**	**Control (n=20)**	
**Age (year)**		56.91±12.15	61.9±11.37	**0.209**^a^
**Sex**	**Female**	8 (35)	10 (50)	**0.313**^b^
	**Male**	15 (65)	10 (50)	
**BMI (kg/m^2-1^)**		24.1±9.4	26.06±10.8	**0.078**^a^
**ASA**	**II**	11 (48)	14 (70)	**0.146**^a^
	**III**	12 (52)	6 (30)	
**Comorbidity**	** DM**	4 (17)	4 (20)	**1.000**^c^
	** Asthma/COPD**	1 (4)	0 (0)	**1.000**^c^
	** Hipertension**	5 (21.7)	4 (20)	**1.000**^c^
**Duration of Anaesthesia (h)**		3.74±1.02	3.63±1.15	**0.666**^a^
**Duration of Surgery (h)**		3.3±0.94	3.09±1.1	**0.513**^a^
**Volume of cristalloid (mL)**		3543.48±928.26	3125±792.65	**0.152**^a^
**Blood Products (packet)**	**ES**	5 (21.7)	3 (15.0)	**0.704**^c^
	**FFP**	1 (4.3)	0 (0.0)	**1.000**^c^
**Volume of colloid (mL)**		413.04±245.51	400.00±261.57	**0.852**^a^
**Rescue analgesic in PACU**		0 (0)	0 (0)	**NA**
-				**n (%)**
**Type of surgery**		**Distal pancretectomy**		1 (2.3)
		**Gastrectomy**		3 (7)
		**Cystectomy**		2 (4.7)
		**Metastasectomy**		2 (4.7)
		**Right hemicolectomy**		4 (9.3)
		**Left hemicolectomy**		5 (11.6)
		**Subtotal pancreatectomy**		1 (2.3)
-		**Total colectomy**		2 (4.7)
-		**Whipple**		23 (53.4)

^a^Mann-Whitney U test,^ b^Pearson chi-square test,^ c^Fisher’s exact test

Summary statistics are reported as medians (interquartile ranges), means ± standard deviations or numbers (%).M-TAPA, modified thoracoabdominal nerve block through the pericondrial approach; SSI, surgical site infiltration; BMI, body mass index; ASA, American Society of Anesthesiology; DM, diabetes mellitus; COPD, chronic obstructive pulmonary disease; ES, erythrocyte suspension; FFP, fresh frozen plasma; PACU: post anaesthesia care unit; NA: not applicable.

**Table 2. Postoperative Morphine Consumption:** 

-		**Analgesia group**		**95% CI of the difference**	***P*^a^**
		**M-TAPA (n=23)**	**Control (n=20)**		
**0^th^-2^nd^ hour**	**Median (Q1-Q3)**	2 (2-3)	4 (4-5)	**-2.72 -0.93**	<0.001^*^
**2^nd^-6^th^ hour**	**Median (Q1-Q3)**	5 (4-7)	6 (4.5-7.5)	**-1.55 2.26**	**0.572**
**6^th^-12^th^** **hour**	**Median (Q1-Q3)**	5 (4-6)	7 (5-8)	**-2.99 -0.32**	0.007^*^
**12^th^-24^th^ hour**	**Median (Q1-Q3)**	4 (2-5.5)	7 (5.5-9.5)	**-4.95 -1.15**	0.001^*^
**24^th^-48**^th^ **hour**	**Median (Q1 - Q3)**	6 (5-8)	25 (19.5-30)	**-23.01 -12.78**	<0.001^*^
**24^th^ hour total**	**Median (Q1 - Q3)**	16 (14-18)	24.5 (19.5-27)	**-9. 42 -3.01**	<0.001^*^

^a^Mann-Whitney U test (Bonferroni correction applied, *P*=0.01)

Summary statistics are reported as medians (interquartile ranges). CI, confidence interval; M-TAPA, modified thoracoabdominal nerve block through the perichondrial approach; SSI, surgical site infiltration; NRS, numeric rating scale.

**Table 3. Numerical Rating Scale (NRS) Scores:** 

-		**Analgesia group**		**95% CI of the difference**	***P*^a^**
		**M-TAPA (n=23)**	**Control (n=20)**		
**0**^th^** hour**	**Median (Q1-Q3)**	3 (2-4)	3 (3-4)	**-0.99 0.15**	**0.205**
**2**^th^** hour**	**Median (Q1-Q3)**	2 (2-2)	3 (2-4)	**-1.63 -0.54**	<0.001^*^
**6**^th ^**hour**	**Median (Q1-Q3)**	3 (2-4)	4 (3-4)	**-1.54 -0.48**	0.002^*^
**12**^th^** hour**	**Median (Q1-Q3)**	2 (1-3)	4 (3.5-4)	**-2.12 -0.93**	<0.001^*^
**12**^th^** hour**	**Median (Q1-Q3)**	1 (0-2)	2 (2-3)	**-2.05 -0.85**	0.001^*^
**24**^th^** hour**	**Median (Q1-Q3)**	0 (0-1)	1 (0-1)	**-0.78 0.58**	**0.116**

^a^Mann-Whitney U test (Bonferroni correction applied, *P*=0.01)

^*^P < 0.05

Summary statistics are reported as medians (interquartile ranges).

CI, confidence interval; M-TAPA, Modified thoracoabdominal nerve block through the pericondrial approach; SSI, Surgical site infiltration.

**Table 4. Need for Rescue Analgesics:** 

-		**Analgesia group**		***P*^b^**
		**M-TAPA (n=23)**	**Control (n=20)**	
**2**^th^** hour**	**Absent**	21 (92)	14 (70)	**0.073**
	**Present**	2 (8)	6 (30)	
**6**^th^ **hour**	**Absent**	19 (83)	11(55)	**0.056**
	**Present**	4 (17)	9 (45)	
**12**^th^** hour**	**Absent**	22 (96)	16 (80)	**0.110**
	**Present**	1 (4)	4 (20)	
**24**^th^ **hour**	**Absent**	23 (100)	18 (90)	**0.120**
	**Present**	0 (0)	2 (10)	
**48**^th^** hour**	**Absent**	23 (100)	20 (100)	**NA**
	**Present**	0 (0)	0 (0)	

^b^Cochran’s Q test. Summary statistics are reported as numbers (percentiles).

**Table 5. Postoperative Patient Satisfaction:** 

-		**Analgesia group**		***P*^c^**
		**M-TAPA (n=23)**	**Control (n=20)**	
**2**^th^** hour**	**Median (Min.-Max.)**	2 (0-3)	2 (0-3)	0.002^*^
**6**^th^ **hour**	**Median (Min.-Max.)**	3 (0-3)	2 (0-3)	0.003^*^
**12**^th^** hour**	**Median (Min.-Max.)**	3 (2-3)	2 (1-3)	0.003^*^
**24**^th ^**hour**	**Median (Min.-Max.)**	3 (2-3)	2 (1-3)	0.012^*^
**48**^th^** hour**	**Median (Min.-Max.)**	3 (2-3)	2 (1-3)	0.001^*^

^c^Mann-Whitney U test. Summary statistics are reported as the median (minimum-maximum).

^*^P < 0.05

M-TAPA, Modified thoracoabdominal nerve block with a perichondrial approach; Min.-Max., minimum-maximum.
